# Exploring perceptions of digital patient reported outcome measures: a multi-site qualitative case study in four European oncology outpatient clinics

**DOI:** 10.1186/s12955-026-02574-0

**Published:** 2026-06-19

**Authors:** Kathrin Cresswell, An Jacobs, Anne-Lore Scherrens, Lise Rosquin, Julien Antonio Luyten, Elias David Lundereng, Victoria Freitas-Durks, Lorraine Warrington, Tonje Lundeby, Marianne Jensen Hjermstad, Robin Williams, Kim Beernaert, Nicoleta Mitrea, Geana Paula Kurita, Marie Fallon, Stein Kaasa

**Affiliations:** 1 Usher Institute, The University of Edinburgh, Edinburgh, UK; 2 Imec-SMIT Research Group, Department of Media and Communication Studies, Vrije Universiteit Brussel, Brussels, Belgium; 3 End-of-Life Care Research Group, Vrije Universiteit Brussel (VUB) and Ghent University, Brussels, Belgium; 4 Department of Surgery, Maastricht University Medical Center, Maastricht, The Netherlands; 5 European Palliative Care Research Centre (PRC), Department of Oncology, Oslo University Hospital, and Institute of Clinical Medicine, Oslo, Norway; 6 INCLIVA Biomedical Research Institute, Valencia, Spain; 7 Leeds Institute of Medical Research at St James’s, University of Leeds, Leeds, UK; 8 Institute for the Study of Science, Technology and Innovation, The University of Edinburgh, Edinburgh, UK; 9 Department of Fundamental Disciplines and Clinical Prevention, Faculty of Medicine, University of Transilvania, Brasov, Romania; 10 Department for Education and National Development, Hospice Casa Sperantei, Brașov, Romania; 11 Multidisciplinary Pain Centre, Department of Anaesthesiology, Pain and Respiratory Support, Neuroscience Centre, Rigshospitalet Copenhagen University Hospital, Copenhagen, Denmark; 12 St. Luke’s Academy of Palliative Medicine, St. Luke’s Foundation, Hellerup, Denmark; 13 Department of Clinical Medicine, Faculty of Health and Medical Sciences, University of Copenhagen, Copenhagen, Denmark; 14 Edinburgh Cancer Research Centre, Institute of Genetics and Cancer (IGC), The University of Edinburgh, Edinburgh, UK

## Abstract

**Background:**

Digital Patient Reported Outcome Measures (PROMs) can help to promote patient-centred care (PCC). However, they are currently not routinely used, potentially compromising patient outcomes. In this work we sought to (i) explore existing processes, patient and healthcare professionals’ (HCP) perceptions of current gaps in PCC and (ii) their views on how a PROMs-based digital system could help to address these gaps in four European oncology outpatient clinics.

**Methods:**

We conducted a qualitative multi-site case study including healthcare staff (organisational leaders, managers and HCPs), patients being treated for cancer and caregivers in four outpatient clinics in Brussels (Belgium), Edinburgh (United Kingdom), Oslo (Norway), and Valencia (Spain). Data were collected through a series of semi-structured interviews to explore existing work practices, needs and attitudes. We also conducted non-participant observations of staff meetings and clinic activities to explore existing processes. Data were analysed through a mixture of inductive and deductive approaches drawing on the Technology, People, Organizations, and Macroenvironmental (TPOM) factors framework.

**Results:**

We conducted 99 interviews with HCPs, patients and caregivers and 30 observations across the four sites. PCC was regarded as important across all sites. We observed limited existing efforts on systematically recording psychosocial needs of patients. Participants reported concerns that a new digital system to record PROMs may result in increased workloads for clinical staff and adversely impact patient-clinician relationships. Attitudes were influenced by previous experience with digital systems. Organisational leadership and support were viewed as crucial in facilitating adoption, including efforts to train and engage clinical and patient users, making available sufficient resources, and including end-users in system design.

**Conclusions:**

While digital PROMs have the potential to enhance PCC in cancer, their routine use is often hindered by sociotechnical challenges. This issue persists across different countries. Success in developing and implementing digital PROMs will require tailored system design and implementation strategies being cognisant of various stakeholder needs. This may include supplementing technological aspects of interventions with educational strategies, supporting local adaptations of designs, and aligning with clinician and organisational drivers for implementation.

**Supplementary Information:**

The online version contains supplementary material available at https://doi.org/10.1186/s12955-026-02574-0.

## Introduction

Cancer remains a major global health burden, with an estimated 18.5 million new cases and 10.4 million deaths worldwide in 2023, and new cancer cases in Europe increasing by 58% between 1995 and 2022 to 3.2 million [1, 2]. Despite improvements in prognosis and outcomes for people with cancer, a diagnosis and associated treatments can have a significant impact on quality of life (QoL) throughout the cancer trajectory. Physical symptoms and treatment side effects can include fatigue, pain, urinary/bowel dysfunction, and mobility problems, while psychosocial issues can encompass anxiety, depression, relationship issues and financial concerns [3–5]. 

Patient-reported outcome measures (PROMs) are defined as “any report of the status of a patient’s health condition that comes directly from the patient, without interpretation of the patient’s response by a clinician or anyone else” [6]. As cancer care increasingly evolves from a predominantly tumour-focused model to a more patient-centred approach that prioritizes improving QoL, PROMs have become an essential resource for capturing patients’ perspectives and lived experiences. By systematically collecting this information, PROMs are considered to be particularly useful for promoting and enhancing patient-centred care (PCC), by placing the patient voice at the centre (e.g. through shared decision-making and by incorporating individual needs and preferences) [7, 8]. 

This study draws on a broad conceptualisation of PCC that emphasises: (i) understanding patients’ individual needs, preferences, and values; (ii) supporting holistic care, including psychosocial dimensions; and (iii) enabling patient involvement in care and decision-making [9]. In line with this, our study does not treat PROMs as outcomes of PCC per se, but rather as potential mechanisms or tools to support and operationalise PCC, particularly by systematically capturing patient-reported symptoms, concerns, and priorities that may otherwise remain unaddressed in routine care.

Technological advances such as increased computer and smartphone usage have made routine and remote collection of digital PROMs viable and more accessible [10, 11]. Patients can complete digital PROMs from their own devices, or devices provided by their care provider, in order for the data to be available to their clinical teams. There is growing evidence for the benefits of this approach, with demonstrated improvements in symptom control, QoL, self-efficacy, communication, unplanned hospital admissions and survival [12–18]. However, despite this accumulating body of evidence and the recent publication of guidelines by the European Society for Medical Oncology (ESMO) [19], digital PROMs are not routinely implemented into cancer care in a standardised way.

Underlying reasons include lack of integration of remotely collected data into existing information technology (IT) infrastructure, a lack of time and resources to support administrative tasks required to register and manage patients, and lack of clear guidance of how to interpret and manage identified problems [20, 21]. In addition, there are concerns about the accessibility and suitability of digital PROMs for certain patient groups (e.g. people facing digital barriers), as well as about the potential burden these tools may place on patients [22]. Overcoming these barriers requires mapping of existing work processes and needs, to tailor both the digital PROMs platform itself and the implementation strategy to each individual clinical context and patient group [23]. 

As part of the MyPath European project, which aims to integrate a digital intervention combining digital PROMs with linked management options in cancer care, this study sought to (i) explore existing processes and gaps in PCC, and (ii) examine perceptions of how a PROMs-based digital system could address these gaps in PCC across four oncology outpatient clinics in Europe [24]. 

## Methods

MyPath is a Horizon Europe funded programme aiming to develop a novel digital PROMs platform with digital support and implement it into nine different clinical sites [24]. MyPath will need to be flexible and adaptable to meet the needs of each site and cancer patient group, to integrate with local IT infrastructure, and to provide guidance on interpretation and management of digital PROMs in accordance with local guidelines and practices. It will also include tailormade pathways based on PROMs. This work forms part of the formative evaluation phase of the MyPath programme.

**Fig. 1:**
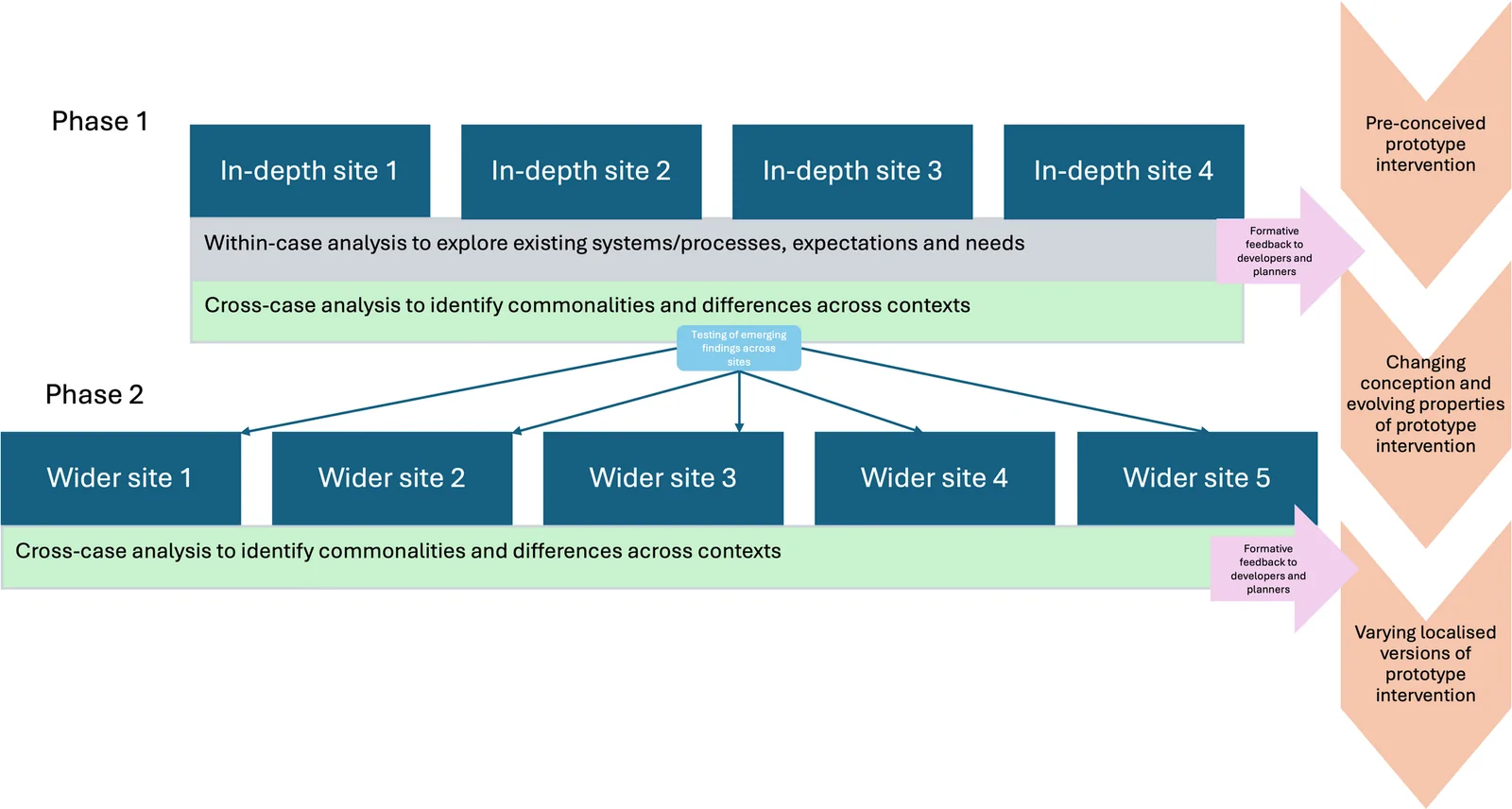
Overview of pre-implementation study

To improve the understanding of the clinical readiness for routine use of digital PROMs at MyPath sites, we conducted qualitative case studies of four different healthcare contexts. In this paper, we explore existing processes and gaps PCC, and how a PROMs-based digital system could address these gaps (Fig. 1, Phase 1). The results of this work will inform the implementation strategy for all the nine sites (Fig. 1 Phase 2). Findings from each case study site will be reported separately in follow-on papers.

We conducted a multi-site qualitative case study between October 2022 and February 2024, collecting data through semi-structured interviews with patients and healthcare staff, and non-participant general observations in the clinical setting under study. The approach was informed by established case study methodology [25, 26], enabling in-depth examination of implementation baselines across different contexts.

Reporting of this study was informed by the Consolidated Criteria for Reporting Qualitative Research (COREQ) guidelines to enhance transparency and methodological rigour [27]. 

### Sampling of case study sites and participants

We selected four out of nine different European project sites involved in the EU MyPath project: Brussels (Belgium), Edinburgh (United Kingdom), Oslo (Norway), and Valencia (Spain). In these sites, we collected in-depth qualitative information about the existing processes and the views of a PROMs-based digital system. Sampling considerations included maximum variation in relation to geographical diversity, range of clinical settings, cancer types, health system set-up, existence of clinical champions, and team sizes. Each site had at least one social scientist in the local team, trained in qualitative research.

Within sites, we conducted a detailed stakeholder mapping to understand existing teams and roles. We mapped the oncology service structure and key people involved in providing oncology care within sites. This included people who were involved in the first appointment, including all surgical and non-surgical treatments, post-treatment oncology care, and allied healthcare staff. We purposefully sampled a range of HCPs and patients from a variety of professional backgrounds and with a range of levels of seniority to get a rounded understanding of different viewpoints. Patients sampled included varying demographics, and different stages and trajectories of disease.

Participants were recruited with the help of local gatekeepers, including clinical leads, managers, and principal investigators. Initial contact was typically made via email through these gatekeepers, with some variation across sites depending on local organisational structures and researcher embeddedness. Patients and caregivers were recruited via their responsible clinicians. All potential participants received a study information sheet and consent form and were given time to consider participation before providing written informed consent. Gatekeepers did not share participant contact details with the research team. Instead, eligible individuals were informed about the study by clinical staff and were invited to contact the research team directly if interested in participating.

We employed a purposive sampling strategy to capture a diverse range of perspectives across stakeholder groups, including patients, caregivers, HCPs, and organisational staff involved in oncology care. Sampling aimed to achieve maximum variation within and between sites in terms of clinical context, organisational structures, and levels of experience with PROMs. The number of participants recruited at each site was influenced by practical considerations, including site size, availability of staff and patients, levels of engagement with the study, and competing clinical priorities. As this work formed part of a formative pre-implementation phase exploring perceptions of a proposed digital intervention, recruitment was also shaped by participants’ willingness to engage with a system that was not yet implemented in practice.

We aimed to recruit as many participants as feasible within the study timeframe while ensuring representation across key stakeholder groups. Although data saturation may not have been fully achieved within individual sites, the overall sample across multiple sites allowed for identification of common themes and cross-contextual variation. Full details of participant numbers and characteristics are provided in the Results section.

### Data collection

Data collection took place between January 2023 and December 2023. We collected data through semi-structured interviews with patients, caregivers and HCPs, as well as through non-participant observations of clinic activities and staff meetings.

Interviews explored general views on PCC, use of current digital systems, and perceived needs and opportunities to draw on digital systems to improve PCC.

Observations were conducted across a range of settings, including multidisciplinary team meetings, clinical environments, and managerial or planning discussions. The specific focus and nature of observations varied across sites, reflecting differences in organisational structures, local implementation contexts, and levels of engagement with the study.

Observations were conducted with researchers adopting an observer-as-outsider role. In line with established observational typologies, this approach is closest to passive participation, where the researcher is present in the setting but does not actively engage in clinical activities or interactions. This positioning enabled the capture of naturally occurring workflows, communication patterns, and care processes within multidisciplinary meetings and clinical environments, while minimising disruption to routine practice. The approach draws on established methodological literature describing degrees of participation in observational research, including the continuum outlined by Spradley and subsequent qualitative research guidance [28, 29]. 

Observations were conducted by one researcher at a time in each site, who documented field notes focusing on care pathways, team interactions, decision-making processes, and the integration of existing digital systems into practice (see Table 2). This approach was chosen to ensure consistency across sites, reduce observer influence, and support comparative analysis of organisational processes in different healthcare contexts. Observational data were used to complement interview data. Rather than serving as a standalone data source, observations provided contextual insight into how care processes, multidisciplinary interactions, and organisational practices were enacted in practice. This enabled triangulation of reported and observed behaviours and supported interpretation of site-specific dynamics.

Observations and interviews were conducted in the local language by lead case study researchers (VFD, EDL, ALS), transcribed, anonymised, and translated for the purposes of analysis. The translations were coordinated locally and validated by local researchers.

### Research material development

Sample interview guides and observation recording sheets are provided in Tables 1 and 2. We did not pilot the topic guides (though we went through several iterations within the extended research team) and we adjusted questions based on the background and circumstances of the individual participant as well as individual contexts. In doing so, we asked HCPs about their experiences of delivering care, whereas we focused patient and caregiver interviews on the processes surrounding receiving care. Topic guides evolved in line with emerging findings, by exploring emerging tensions and opportunities.

Interview guides and observation protocols were informed by a broad conceptualisation of PCC, focusing on current care processes and communication practices, the identification of unmet needs (particularly psychosocial), and perceptions of how digital PROMs could enhance PCC. These tools were not explicitly structured around a single formal PCC model; rather, they were designed to capture multiple dimensions of PCC as they manifest in real-world clinical workflows.

**Table 1:** Sample interview guide

Topic area	Primary questions	Prompts
**Patient-centred care (PCC) – introduction**	Can you describe your experiences of cancer care delivery within your service?	What aspects of care work well? What could be improved? Can you describe an example of patient-centred care in practice? How are patients’ needs (including psychosocial needs) currently identified and addressed?
**Digital systems (transition)**	How are digital systems currently used within your role and service?	What has your experience of using these systems been? What are the main benefits? What challenges or limitations have you encountered? How do these systems affect communication, workflows, or decision-making? What improvements would you suggest?
**Digital outcome measures and care pathways (key focus)**	What is your understanding and experience of using digital PROMs/PREMs, or systematically capturing patient outcomes and experiences?	What potential benefits do you see for patient care? What concerns or challenges do you have (e.g. usability, data burden, trust)? How might PROMs/PREMs influence patient–clinician interactions? How could these tools be integrated into care pathways? How could they be better aligned with patient and service needs?
**Closing**	Is there anything else you would like to add about your experiences or views?	Are there any additional comments or reflections? Are there other individuals you would recommend we speak to?

**Table 2:** Sample observation recording sheet

Location (place and space)
Date
Observer(s)
• General description • Activity(ies) observed
Duration (start/stop time)
Is the activity a one-time event or recurrent? If it is recurrent, what is its frequency?
Participant consent?
Observation (what actually happens)
• Who is participating? • How many people are participating? • What is their role? • What does everyone’s participation consist of?
• How is PCC organised? • How are PCC aspects addressed? • Which IT-solutions/digital systems are used? • How is the patient flow/trajectory organised?
Reflections (research questions)
• What do I see going on here? • What did I learn from these notes in terms of specific barriers/facilitators, current work practices/ infrastructures? • How do I envision that MyPath could be used? Relevance for MyPath? • Why did I include them? • How does my background influence the way I observe things (reflexivity)?
Other things to consider
What else is happening in this site, that might be relevant to my research question(s) and to observations?

### Data analysis

Analysis was conducted between January 2023 and February 2024. Observation notes and transcripts were uploaded into the qualitative research software NVivo 12 (Lumivero). Case study researchers led on each individual case study analysis. We established a cross-case analysis team including all authors. We met monthly from the beginning of data collection to discuss emerging topics and themes. Observational data were integrated with interview data during analysis to provide complementary insights into both reported and observed practices. Observational data were analysed alongside interview data using the same coding framework, contributing to theme development and cross-case comparison.

Data were analysed using thematic analysis following the approach described by Braun and Clarke [30]. Analysis involved cross-case analysis and the use of combined inductive–deductive analytical approaches [31–34]. The deductive element included drawing on the Technology, People, Organisations, and Macroenvironmental factors (TPOM) framework as a coding frame in NVivo for each of the sites as a starting point for coding as a sensitising analytical lens. TPOM is a sociotechnical framework developed to understand the implementation of complex digital health interventions by examining interactions between technological systems, individual users, organisational processes, and wider system-level influences. It is informed by extensive literature on health information technology implementation and sociotechnical systems.

In this study, TPOM was used within a combined inductive–deductive thematic analysis. The framework informed the initial coding structure, enabling data to be organised across its four dimensions, while allowing for the identification of new themes emerging inductively from the data. This approach facilitated a structured yet flexible analysis, supporting both within-case and cross-case comparisons, and enabling exploration of how the implementation of digital PROMs is shaped by interactions between sociotechnical dimensions. Specifically, TPOM provided a structured lens through which to examine how sociotechnical factors influence the implementation of digital PROMs and their potential to support PCC.

As data collection progressed and in line with emerging findings, we adjusted the coding framework based on new insights from the fieldwork. Raw data and NVivo project files remained securely stored at each participating site and were accessible only to local research teams. Initial coding was undertaken locally, after which cross-site analytical discussions were held to compare coding structures, refine themes, and identify patterns across settings. To comply with local governance and data protection requirements, raw data and NVivo project files were not shared between sites.

Regular cross-site meetings were held to discuss emerging findings and promote consistency in interpretation across research teams. In our monthly meetings with social scientists, we discussed the codes that did not fit the framework and detailed the shared meaning of the codes in the framework, adding subdimensions and definitions to be used while coding and recoding the data in the next month. In addition, we noted and discussed emerging similarities and differences between sites.

Towards the end of the data collection (December 2023), we began formulating implications for system design and implementation strategy to inform design of the MyPath intervention going forward.

A completed COREQ checklist is provided in Supplementary Material 1.

## Results

We conducted 99 interviews with HCPs (*n* = 59), managers (*n* = 10), patients (*n* = 19) and caregivers (*n* = 12), and 30 observations across the four sites. Interview durations ranged from approximately 28 to 90 min (mean of 54 min). Interviews were conducted predominantly face-to-face in clinical settings (clinic or hospital). Observation durations ranged from approximately seven to 60 min (mean of 42 min). 

Table 3 provides a detailed description of the case study sites. Data were captured from clinical leads involved in the study.

**Table 3:** Clinical site characterisation

	Brussels	Edinburgh	Oslo	Valencia
**Selected clinical setting based on cancer type **	Lung and prostate cancer	Breast cancer	Testicular, pancreatic, head and neck cancer	Pancreatic cancer
**Type of hospital**,** location (how many centres**,** core or satellite)**	University Hospital, one location	University Hospital, satellite location as initial pilot	University Hospital, comprehensive cancer centre, two locations	University Hospital
**Number of patients per year in oncology for the selected cancer types**	125–130 new lung cancer & 70–90 new prostate cancer diagnoses each year	150 new breast cancer patients per year in satellite centre	450 new head and neck cases per year, 160–170 new testicular cancer cases per year, 25–35 new pancreatic cancer cases per year	90 pancreatic cancers
Oncology department staff* for the selected cancer types	Centre of Oncology (Medical Oncology, Radiation therapy, Supportive and Palliative Care) For the selected cancer types: 7 Medical Oncologists, 4 Oncocoaches, Nursing Team, 3 Social Nurses, 3 Dieticians, 5 Psychologists, 1 Palliative Care Specialist, 1 Psychologist, 3 Physiotherapists, 1 Onco-pharmacist	2 Senior Oncologists, 2 Rotating Trainees,1 Specialty Doctor, 3 Senior Breast Cancer Nurses, 1 Lymphoedema Specialist, shared pool of Physiotherapy, Psychology, Nutrionists, Occupational Therapy and Social Workers with Hub in main cancer centre Cancer Charities provide significant support	Pancreatic: 3 Senior Oncologists, 1–2 Oncologists in Training, 2 Physical Therapists, 2 Social Workers, 1 Psychologist, 1 Nutritionist Testicular: 3 Senior Oncologists, 4 Oncologists in Training, 1 Pathway Coordinator Head and neck: 7 Nurses, 9 Attending Oncologists, and 8 Physicians in Oncology Training Allied health resources available for testicular and head and neck: 10 Physical Therapists, 3 Occupational Therapists, 3 Social Workers, 2 Nutritionists, 2 Psychologists Available across all tumour sites: 1 Sexologist, 1 Psychiatrist, 1 Psychiatric Nurse	16 Oncologists, 1 Head of Department, 1 Oncologist Supervisor, 10 Resident Oncologists, 2 Nurse Supervisors, 30 Nurses, 18 Nurse Auxiliars, 1 Psychologist and 4 Administrative Workers
**Current digital Systems**	Primuz (local electronic health record & patient portal), Radiology Chemotherapy	TrakCare which is used for multiple purposes, Chemocare, PACS (for radiology), MEDIBLE (for ePROS), APEX (blood results)	DIPS (for patient records), MetaVision, CMS (chemotherapy), ARIA (radiation)	Orion clinic (electronic health record), Connectall (electronic system that informs patients have arrived, assigns a code and calls patient in), Oncofarm (chemo prescription), Abucasis (primary care electronic history), Librelink (glucose control)
**Current PROMs in use**	Limited, mostly used in in-patient care at intake (medical, not psychosocial) and systematic pain assessment 3x/day	Pre- and post-operative (paper-based)	Limited in systematic use, ESAS most frequently used, but primarily in palliative care. Other PROMs used for research purposes.	Not used. PROM is retrieved in individualised consultation in a non-systematic way.
**Previously trialled cancer apps for symptoms**	None	Owise	Choice (2008), only trialled for nurses and lymphoma patients	None
Psychosocial support*	In-hospital psychologists from the palliative support team or referral to other psychologists, social workers from the Oncology Centre. Survivorship clinic.	Cancer Treatment Helpline (CTH), Clinical Psychology (referral to Edinburgh Cancer Centre), MacMillan cancer centre (cancer charity), Marie Curie (charity), Maggie’s Centre (charity)	In-hospital psychologists, psychiatric nurses, psychiatrists, social workers, palliative care, spiritual counsellors. The Norwegian cancer society offers a helpline and peer-support groups.	One psychologist from the hospital and another psychologist from an ONG (AECC).

* some of the roles are shared with more than the selected cancer types

Tables 4 and 5 detail the data collected in interviews and observations across sites. Time to diagnosis and age were self-reported by patients.

**Table 4:** Interview participant demographics

Sample characteristics	Brussels	Edinburgh	Oslo	Valencia	Total
**Management**	3	1	5	1	**10**
Male	0	1	0	1	**2**
Female	3	0	5	0	**8**
Mean age in years	35	65	49	66	
Missing	1				
**Healthcare professionals**	**12**	**13**	**24**	**10**	**59**
Male	2	2	8	0	**12 **
Female	10	11	16	10	**47**
Mean years of experience in Oncology	13.7	17.9	16.3	14.9	**15.9**
Mean age in years	40.5	45.5	49.1	41.2	
Missing	1				
**Patients**	**4**	**7**	**3**	**5**	**19**
Male	2	0	2	3	**7**
Female	2	7	1	2	**12**
Mean time since diagnosis in months	Data missing	29.1	8.3	19.4	**21.7**
Mean age in years (mean)	69.5 (60–78)	52 (41–65)	68.3 (63–71)	66.8 (50–79)	
**Caregiver**	**4**	0	6	**1**	**11**
Male	1	0	3	0	**4**
Female	3	0	3	1	**7**
Mean age in years (range)	68.8 (range data missing)	No caregivers interviewed	69.6 (53–78)	46 (range data missing)	
**Total sample**	**23**	**21**	**38**	**17**	**99**

**Table 5:** Observations characteristics

Sample characteristics	Brussels Medical oncology Brussels University Hospital	Edinburgh Breast cancer care	Oslo Head and neck and Testicular cancer, Radium-hospitalet Oslo	Valencia Pancreatic cancer, skin cancer, lung cancer
Dates of observations	31/01/2023–22/06/2023	01/07/23–29/08/23	06/06/2023	21/11/2023–09/01/2024
Number of observations	10	5	10	5
Function/role observed	Medical (Oncologist) & Allied Healthcare professionals	Nurse Specialist	3 Oncologists, 2 in head and neck and 1 in testicular	2 Oncologists
Activity observed	Multidisciplinary Oncology Consultation (hospitalisation) Multidisciplinary Oncology Consultation Urology; Activities at the department of hospitalisation; Consultations oncologist and patients; Outpatient clinic activities	Strategic planning meetings and everyday work	Outpatient clinic for radiation treatment during curative radiation. Also, survivorship follow-up in outpatient clinic	Consultation with patients, both outpatient standard care and clinical trial patients

Our analysis identified three overarching themes: (i) current socio-organisational barriers to delivering PCC, (ii) perceptions of digital PROMs and implementation challenges, and (iii) organisational capacity to support change and adoption. Within these themes, several sub-themes emerged. The first theme captures existing gaps in PCC, including limited systematic recording of psychosocial needs, variation in access to support, and multidisciplinary dynamics. The second theme explores perceptions of digital PROMs, including anticipated benefits as well as concerns relating to usability, data burden, and impacts on clinical interactions. The third theme focuses on enabling conditions for implementation, including organisational support, training, user engagement, and resource availability.

Table 6 illustrates the emerging themes and sub-themes mapped to the TPOM dimensions. These will be discussed in detail in the subsequent paragraphs.

**Table 6:** Overview of emerging themes mapped to the TPOM framework

Overall Theme	Specific Finding	TPOM Dimension(s)	Rationale
Current socio-organisational barriers to delivering PCC	Limited existing efforts of systematically recording psychosocial needs	Organisation	Reflects organisational processes, priorities, and documentation practices within clinical workflows.
	Lack of education of patients and clinicians	People / Organisation	Relates to individual knowledge and skills (People) and organisational provision of training (Organisation).
	Multidisciplinary tensions between oncologists and palliative care	People / Organisation	Reflects professional roles, relationships, and inter-disciplinary coordination within organisational structures.
	Local variations in levels of experience with PROMs	Organisation / Macroenvironment	Influenced by local institutional maturity (Organisation) and broader system-level context (Macroenvironment).
The role of digital tools in delivering PCC	Potential unintended consequences (trust, impact on interactions, data overload)	People / Technology	Relates to user perceptions and cognitive burden (People) and system characteristics such as alerts and data flows (Technology).
	Different digital tools already used by patients and staff	Technology / Organisation	Reflects existing IT infrastructure (Technology) and how these are embedded in workflows (Organisation).
	Previous negative implementation experience	People / Organisation	Influences user attitudes and acceptance (People) and reflects organisational history of implementation (Organisation).
Organisational capacity to support change and adoption	Training and engagement of clinician and patient users	People / Organisation	Focuses on user capability (People) and organisational strategies for engagement (Organisation).
	User involvement in design to improve acceptability	People / Technology	Reflects co-design and usability (Technology) alongside user engagement (People).
	Resources: time and staff	Organisation / Macroenvironment	Reflects organisational capacity and wider system-level constraints such as workforce and funding pressures.

### Current socio-organisational barriers to delivering PCC

#### Limited existing efforts for systematically recording psychosocial needs

PCC was viewed as an important priority across all sites (especially by patients), but we observed limited existing efforts to systematically record the psychosocial needs of patients. Most clinical communication and documentation focused on noting medical history, complications, treatment regimen, and in some instances pain.

For all the patients we operate on, we have to record information about the patients after surgery such as bleeding after the operation or if they need a new operation due to a complication. So, we are more concerned with physical aspects. We are working on incorporating patient-reported outcomes such as pain, well-being, and similar factors because we understand that we also need to focus on these things. But that’s not our primary focus from a surgical perspective. Oslo, Surgeon

Clinicians in Edinburgh explained that they struggle with psychosocial support needing referral and specialist intervention because of the limited specialist resources. Furthermore, charities have been subject to financial constraints and can offer limited practical social and financial support. The desire to help patients after identifying problems is hampered by the macro environment in which clinicians operate.

Observational data supported these findings by illustrating how patient-centred aspects were typically addressed in practice. For example, in observed consultations, clinicians often focused initially on biomedical results and treatment decisions, with patient-centred concerns such as psychosocial symptoms emerging later in the consultation, typically in response to general prompts (e.g. “Do you have any other concerns?”). These concerns were usually raised through patient recall rather than systematic assessment.

While patients frequently reported symptoms such as pain, fatigue, or psychological distress, these were often addressed through reassurance or explanation rather than leading to further exploration or intervention. This suggests that, although PCC was recognised as important, it was not consistently operationalised through structured processes in routine care. 

Patients and clinicians generally had positive attitudes towards digital PROMs.

I would already want to report [problems]. I am in my non-treatment week now, next week is treatment week. Imagine something would happen this week, I would already mention it [in digital PROMs]. This way, the doctor is already notified. And then I also don’t have to burden [nurse]. Brussels, Patient

It could be helpful to check on the app before the consultation how the patient has been feeling and to know where to focus or what to explore. Valencia, Oncologist

In some sites, psychosocial support was viewed as being easier to access than in others. Some patients in Brussels and Valencia explicitly mentioned that they felt adequately taken care of in relation to their psychosocial needs and, therefore, did not feel they needed a new technological system to record these symptoms.

Well, she (the doctor) sees me every week, sometimes Monday and Thursday on the same week, and she asks me everything: about my appetite, about pain… In fact, she asked if I wanted to see a psychologist and I said no, because I didn’t need it so far (…). I see her every week and she already asks me everything, she tells me Monday I will be there (at the hospital), if you need anything… I also have an emergency Oncologist 24 h available… So, for me everything is being taken care of and right now I don’t want to use any apps (…).Valencia, Patient

#### Lack of education of patients and clinicians

Other patients saw the potential of a digital system to record their psychosocial symptoms and saw it as an educational opportunity and as a source of information (Edinburgh, Valencia).

I think it [digital PROMs] sounds like a very good aid, so that when you come for a consultation, both patients and relatives experience that the oncologist is a bit more prepared and has used the tool, and sees the whole picture better. Oslo, Caregiver

HCPs also stated that there was a lack of education in relation to management of psychosocial needs (Edinburgh, Oslo).

Like recently there’s a complete epidemic in Edinburgh of patients on Quinine [antimalarial drug with anti-muscle cramp properties] where the oncologists are taking the muscle cramps at face value, as opposed to being part of neuropathic pain sort of thing. Edinburgh, Nurse Specialist

#### Multidisciplinary tensions between oncologists and palliative care

We also noticed different foci and priorities between oncologists and palliative care specialists. Oncologists were seen as focusing mainly on curative treatments, while palliative care specialists were focused more on addressing psychosocial needs (in Brussels and Edinburgh). In other settings (Valencia), where relationships between professions were more closely linked in practice (e.g. where palliative care was delivered routinely through multi-disciplinary teams working together in the home setting), we did not find evidence of these tensions in either interviews or observations. In this context, both outpatient oncologists and palliative care oncologists offering at-home care indicated that they maintained open channels of communication and a close professional relationship. However, this should not be viewed as definite evidence that these tensions did not exist.

Oncologists paying attention to [psychological wellbeing] facilitates our work, when they say: it might be a good idea to involve [the psychological support team]. (…) And I think the opposite: doctors that are less likely to refer or doctors that are therapeutically persistent. That makes our work difficult. Brussels, Palliative Care

#### Local variations in levels of experience with PROMs

Sites had different levels of experience with PROMs, with some having no prior experience (Valencia) and others having trialled digital PROMs systems but having stopped using these in routine care (Edinburgh, Brussels). Concerns included previous negative implementation experiences (Edinburgh, due to onboarding challenges), doubts regarding whether entered information would be used in practice (Oslo), and time constraints associated with system use (Brussels).

We used to use the Distress Barometer [a tool used to measure levels of distress], I am actually a fan of this, because it gives a good indication of [signs of depression]. The problem is that it is very time-consuming for psychologists and that’s why we stopped using it. Brussels, Psychologist

Nevertheless, there was a general feeling across sites that a digital system could have potential in promoting PCC.

### Perceptions of digital PROMs and implementation challenges

#### Potential unintended consequences: trust, adverse impact on interactions with patients, data overload

HCPs across sites expressed concerns that a new digital system to record PROMs would result in increased workloads. HCPs reported already using multiple general digital systems across sites and whilst they saw the benefits of a systematic approach to promote PCC through PROMs, they also expressed concern surrounding additional problems that a digital system would uncover that would then have to be addressed in time limited consultations.

And the basic principle is based on the fact that all oncologists want to do their very best for patients, (…) but the reality is, with the increasing armamentarium of potential treatments and investigations etc. and the amount of information that has to be conveyed to patients (…) on quite a regular basis from the point of diagnosis (…) it’s increasing difficult one would imagine with in the standard consultation time to deal with other issues (…) if a patient has very severe pain then that might (…) come out in the consult, but (…) a lot of other problems won’t. Edinburgh, Oncologist

Observational insights also highlighted the current balance between clinical focus and patient engagement. Clinicians appeared to prioritise direct interaction with patients and spent limited time engaging with digital systems during consultations. While this supported patient–clinician rapport, it also suggests that the introduction of additional digital tools would need to be carefully integrated to avoid disrupting these interactions or increasing cognitive and workflow burden.

What I don’t like is that during consultation, more often than not, I need to use so many apps that I loose time for [attending] to the patient, of looking at them face-to-face. Valencia, Oncologist

Some also expressed concerns regarding trust in any new digital system including potential overreliance on it (Oslo, Brussels) and expressed worries that the use of digital technology may adversely impact on interactions between HCPs and patients (Brussels, Valencia). The main concern in Edinburgh was past experience with poor technology in relation to onboarding, which lead to a significant waste of time for both patients and staff.

We need appropriate education on all problems which may be highlighted by e[lectronic]PROMs either in relation to management or appropriate onward referral Edinburgh, Oncologist

At the end you have so many apps open that it takes time away from looking at the patients’ eye Valencia, Oncologist

Some also reported a fear of potential data overload resulting from the introduction of a new technological system including patient-generated data (Edinburgh, Brussels), whilst in contrast others reported wanting more information (Brussels). In Brussels, this belief differed across HCP’s roles, with some wanting more information than others (e.g. nutritionists). Wanting more information was seen as particularly important for specific patients that would not freely share their symptoms during the consultation (because they were time pressured, had delayed symptoms or did not want to worry a caregiver attending with them).

I need to bite my tongue when we see her oncologist, “it’s all good” she says when it is not. Brussels, Caregiver

#### Previous negative experiences with digital tools

Previous experiences of digital technology impacted attitudes towards the use of a new system recording PROMs. We observed differences in the levels of digital tools already used by patients and staff across different settings. Some of these included negative experiences with prior implementation of technological systems, and instances where these were perceived to lack usability and usefulness (Edinburgh, Brussels, Oslo).

Especially the CMS [clinical management system] that is now being phased out. It was never a success. I don’t think anyone saw it as helpful. Quite the opposite. [Other system] is similar. People aren’t excited about it, but it had to come. It was supposed to be an electronic curve, and everyone understands that, but the software isn’t good. This breeds scepticism. It’s natural. It’s yet another thing coming, so from past experience, no one is applauding in advance. It makes people more cautious and wait to see what comes next. Oslo, Oncologist

Clinical decision support systems involving alerts were generally viewed as having inherent dangers due to alert fatigue and oncologists favoured individual assessment with linked management options. HCPs also highlighted the need to consider individual patient circumstances which decision support systems may not be able to account for (Edinburgh, Brussels).

Not every patient fits in a box. (…) Maybe this works with 60 to 70% of patients, if it’s very straightforward, but with a number of patients, automatically developing a treatment plan won’t work. Brussels, Manager

### Organisational capacity to support change and adoption

#### Training and engagement of clinician and patient users

Organisational factors associated with a new digital system incorporating PROMs were viewed as important across sites, as these could either facilitate or hinder implementation. In particular, participants highlighted the importance of clinician engagement and the role of early adopters in promoting acceptance of the system. Several participants suggested that involving motivated clinicians from the outset could help demonstrate the value of the system to colleagues and encourage wider uptake.

Maybe if we start with few clinicians, those who want to try it, they might speak out about the benefits of the app. For example, in the case of pancreatic cancer, they are usually very symptomatic patients, and you have a lot of areas to evaluate. If the patients answer those questions before coming to the consultation, you may already know which are the problematic areas and you have a place to start. Valencia, Oncologist

Participants also highlighted the importance of receiving training to promote standardised use of the system, expressing concerns that inconsistent implementation could undermine both the perceived benefits of digital PROMs and the ability to evaluate their effectiveness.

You’d have to give some education on what to do with it. Usually. Otherwise, everyone will use it in a different way so I think there would have to be training to make sure we’re all using it in the same way, otherwise the benefits from it will be different, depending on how you’re using it. And then it’ll be difficult to interpret how useful it’s been if everyone’s using it in a different way. Edinburgh, Registrar

#### User involvement in design to improve acceptability

Others mentioned the crucial role of organisational management in introducing changes accompanying the introduction of technology, including training of clinicians and patient users (Valencia, Brussels), ongoing engagement and involvement of clinical users (Brussels, Valencia, Oslo, Edinburgh), active user involvement in system design to improve acceptability, and allocation of adequate resources including additional time and staff to manage changes (Oslo, Valencia).

For me to be able to trust and want to use such a solution [digital PROMs], something must happen when you report symptoms. Otherwise, it’s just fake, and that’s almost worse than not being asked in the first place. Oslo, Caregiver

Some also mentioned that feedback to clinicians as to how a system improved their clinical performance would be valuable for their motivation to use it and that tailoring a system to local needs is important.

#### Resources: time and staff

We observed differing views across sites in relation to the perceived responsibility for recording and acting on system outputs e.g. alerts (Oslo), coordination across specialties (Brussels), and information required by different stakeholder groups (Edinburgh). For example, HCPs stated that administrative staff would need to explain a new tool to patients and to save time during the consultation, but this would have resourcing implications.

Sometimes there are 5-minute long consultations and there is no need for more [time] because everything is fine. Other times you need 25 [minutes] in a consultation because there is a change in the treatment, some bad news [to be given]. Having those 25 minutes is something both clinicians and patients would appreciate. And sometimes I would like to have a bit more. Valencia, Oncologist

Some also mentioned that HCPs would need to be presented with information that is relevant to their specific role. In all sites, the oncologist was perceived as the key HCP responsible for following up on digital PROMs. However, in Brussels, the “oncocoach” - a nurse specialized in oncological care - was also perceived as key in managing the digital PROMs.

The variability in observed practices across sites (including differences in consultation structure, communication styles, and integration of patient-centred elements) also reflects differing levels of organisational readiness for change. These differences highlight the importance of tailoring implementation strategies to local contexts.

## Discussion

### Summary of main findings

Despite differences in health systems and cancer settings, it was acknowledged across all four sites and across stakeholder groups that PCC is crucial, but a systematic approach to PCC is often lacking. Regarding processes, there was limited education surrounding PCC for patients and clinicians, and there were in some instances differences in attitudes towards care for patients with potential palliative care needs (medical care focus versus psychosocial care focus). HCPs voiced major concerns about a new digital system for recording PROMs potentially increasing workloads although some hoped it could improve efficiency. Issues around trust in digital systems, potential information overload, and previous negative experiences with technology also posed barriers to adoption. Implementing digital PROMs requires strong organisational support, adequate training, user engagement, and coordination among specialties.

### Integration of findings with the existing literature

There is clear perceived potential and well acknowledged evidence from controlled clinical trials for using digitally-enabled PCC [13, 35, 36]. However, despite this, digital PROMs have not been successfully embedded in routine care at scale [37]. Underlying reasons include difficulties aligning various stakeholder needs, information technology and infrastructure related issues, and disruption to clinical work routines [38–40]. There is also a large body of literature surrounding decision support systems and the risks of users developing alert fatigue (although PROMs do not always have to have a clinical decision support element) [41]. These have been supported by our findings where clinical stakeholders worried that digital PROMs would have adverse impacts on workloads. This was especially the case in settings that had previous negative experiences with technology implementation and where systems did not align with existing practices [42]. 

Implementing evidence-based practice into routine practice is challenging, especially in multi-disciplinary settings with complex existing practices and infrastructures [13, 35, 43]. As such, the use of routine digital PROMs in oncology practice will require a change in working behaviour for HCPs and organisations [39, 44]. These aspects are all reflected in the findings of this study, calling for training of staff as well as involvement and commitment from organisational leaders.

New models for cancer care need to be developed to coordinate and plan these changes. The concept of “innovation readiness”, an organization’s maturity and capacity to implement innovations, can help to inform such efforts. Factors that have been identified to play a role include having a clear innovation strategy, fostering an environment that supports innovations, engaging leadership, and an organisational commitment to innovation [45]. 

Our work has added to this literature is that: (1) these issues appear to be independent of health system context as we observed them across case studies of several countries; (2) an understanding that some of these issues may be addressed through education of clinicians and patients, clear outlining of expected benefits, as well as through managing multidisciplinary tensions; and (3) innovation readiness is an important factor for the adoption of digital PROMs.

Organisational dimensions such as leadership and support for digital interventions are well-known to be crucial in promoting implementation and adoption [46]. This needs to include targeted education of clinicians and patients, making available necessary resources to support the adoption of PROMs, and promote user involvement in their design. Understanding individual organisational drivers for implementation in individual sites is therefore critical.

An important consideration emerging from this work is the role of PROMs data itself as a potential driver of adoption and sustained use. While participants frequently discussed challenges related to workload, system usability, and organisational readiness, there was comparatively less emphasis on how PROMs data would be used in practice to inform care. This may reflect the pre-implementation nature of the study, where participants were engaging with a proposed system rather than an established intervention with demonstrated benefits. However, existing literature suggests that the perceived value of PROMs data (particularly its ability to inform individual patient management, support shared decision-making, monitor treatment outcomes, and contribute to service improvement) is a key facilitator of successful adoption [47–49]. Ensuring that PROMs data are actionable, meaningfully integrated into clinical workflows, and clearly linked to patient benefit will therefore be critical. This includes providing feedback to both clinicians and patients on how data are used and demonstrating tangible impacts on care. Without this, digital PROMs risk being perceived as an additional administrative burden rather than a tool that enhances PCC. Future work should therefore prioritise not only the technical implementation of digital PROMs, but also the development of clear pathways for how collected data are interpreted, acted upon, and fed back into clinical and organisational decision-making.

While this study primarily identified sociotechnical and organisational barriers to implementing digital PROMs, it is important to more explicitly consider their potential to enhance PCC. PROMs offer a mechanism to systematically capture the patient voice, including symptoms, concerns, and priorities that may otherwise remain unrecognised within routine consultations. In doing so, they have the potential to support more personalised care, facilitate shared decision-making, and enable more proactive management of patient needs. The relatively limited emphasis on these patient-centred benefits within our findings may reflect the pre-implementation nature of the study, where participants were asked to reflect on a proposed system rather than one experienced in practice. As such, participants may have found it easier to articulate anticipated challenges than to envisage how PROMs data might be meaningfully integrated into care. Future phases of this work will explore in greater depth how the use of PROMs in practice influences PCC, including how data are used in clinical encounters and how patients perceive their value.

Importantly, there is also a need to consider the potential for unintended consequences associated with digital PROMs implementation. Previous research on digital health interventions has demonstrated that technologies designed to improve efficiency, communication, and patient-centred care can also generate unforeseen effects, including increased workload, alert fatigue, workflow disruption, and the exacerbation of existing inequalities among groups with lower digital literacy or access to technology [50–52]. Our findings suggest that similar concerns exist among both patients and healthcare professionals in oncology settings. Rather than representing barriers alone, these concerns identify important areas for ongoing evaluation and optimisation during and after implementation. Future research should therefore examine not only whether digital PROMs improve patient-centred care, but also how they influence clinical workflows, professional roles, patient experiences, and equity of access across different healthcare settings. Such work will be essential to ensure that digital PROMs enhance rather than inadvertently undermine PCC.

### Implications for policy and practice

This work will form the basis for developing and tailoring a digital PROMs system to promote oncology PCC in nine European cancer centres. As such, findings will feed into system development and local system configuration, as well as central and local implementation strategies. We will also seek more information along the way during implementation to optimise processes and reduce barriers to adoption.

Key strategic considerations to ensure maximum chances of successful adoption before implementation include exploring and aligning trade-offs and compromises associated with system implementation for different stakeholder groups, planning for social as well as technological aspects of digitalisation (e.g. changes in work practices), and considering organisational drivers for supporting system development and implementation. Organisational dimensions are likely to include integration and interfacing work required to effectively embed the new system. In some instances, there may be a need to shift from designing a new system to extending existing functionality in sites.

Starting with a limited scope may help to achieve stakeholder alignment. It may also help to allow for different conceptions of the system and associated changes to be developed and/or implemented in different sites. This will in turn facilitate local ownership and promote engagement.

Most importantly, it is vital to recognise that any complex intervention with technological components will also always require behavioural changes. Educational components for both patient and clinical users are therefore crucial. This should involve clear communication of the value of digital PROMs, particularly those addressing psychosocial aspects. Furthermore, this educational component needs to be reinforced by the technology itself, whilst seeking to avoid technology-driven implementation strategies.

Applying the TPOM framework to interpret our findings highlights that the implementation of digital PROMs is inherently sociotechnical and shaped by interdependent factors across multiple dimensions. Many of the issues identified in this study (e.g. concerns about workload, data burden, trust in digital systems, and variability in adoption across sites) mirror those reported in the implementation of other complex digital health interventions [15, 43, 44]. Importantly, these challenges are rarely attributable to technology alone, but arise from misalignments between technological systems, organisational workflows, user expectations, and resource constraints. The TPOM framework also draws attention to the role of macroenvironmental factors, including differences in health system structures, policy contexts, and resource availability, which are likely to influence how digital PROMs are adopted, adapted, and scaled across settings. These will likely increasingly come into play as implementation progresses.

### Strengths and limitations

We collected a large qualitative dataset across multiple settings and stakeholder groups, providing insights into common drivers, challenges, and contextual variation in relation to PCC and the potential implementation of digital PROMs. The use of both interviews and observations enabled us to capture complementary perspectives on reported and enacted practices. However, the findings presented here reflect a formative, cross-site analysis, and do not yet provide the level of granularity required to fully tailor PROMs implementation to specific disease areas, stakeholder groups, or organisational contexts.

Rather than offering definitive solutions, this work identifies key sociotechnical considerations that are likely to influence implementation across settings. Further, more detailed analyses (conducted alongside ongoing system development and implementation within the MyPath programme) will be required to generate more fine-grained, context-specific insights. As such, the current findings should be interpreted as providing a foundation for subsequent phases of work, rather than a complete account of how PROMs systems can be optimally configured for all settings.

There are also potential challenges in comparability across study settings, as variations in practices, cultures, and languages may have influenced findings. For example, there were variabilities in interview responses from professionals both within and across sites. This may affect the viewpoints expressed. In addition, the findings may not be applicable to other oncology specialties such as surgical oncology. Our sample also reveals a gender imbalance amongst HCPs, with many more females than males participating (47 compared to 12), reflecting the predominance of female HCPs in oncology.

This study involved data collection and analysis across multiple countries and healthcare settings, which introduces potential variability related to cultural, organisational, and researcher differences. While efforts were made to support consistency (through the use of shared data collection tools, regular cross-site analytical discussions, and a common coding framework) data were generated and interpreted within local contexts. This may have influenced how concepts such as PCC and digital system use were understood and articulated. However, rather than representing solely a limitation, this variation also reflects the real-world complexity of implementing digital health interventions across diverse settings. Preserving sensitivity to local context is important, particularly given the aim of developing a system that can be adapted to different healthcare environments. The TPOM framework provided a structured lens to support comparison across sites, while still allowing for context-specific insights to emerge. Future work within the programme will build on these findings by developing more detailed, contextually grounded case study narratives and implementation strategies.

An additional limitation relates to variation across disease areas and clinical contexts included in this study. Participating sites differed not only in organisational structures and practices, but also in the types of cancer and stages of disease represented. For example, some settings focused on conditions with more advanced or palliative trajectories (e.g. pancreatic cancer), where immediate symptom management and supportive care needs may be prioritised, while others involved patient groups with longer-term treatment and follow-up pathways. These differences are likely to influence both the conceptualisation and delivery of PCC, as well as perceptions of the potential value and use of digital PROMs. As a result, direct comparisons across sites should be interpreted with caution, and findings may not be equally transferable across all oncology contexts. Future work should explore these variations in greater depth to inform more tailored implementation strategies.

Although interview guides were developed and refined by a multidisciplinary research team, they were not formally piloted with patients or caregivers prior to data collection. Formal piloting may have further enhanced accessibility and ensured that questions elicited the intended information from all participant groups.

## Conclusion

Although there is a clear drive and potential for digital PROMs in facilitating oncology PCC, the routine use of systems is hampered by sociotechnical dimenisons of change. This appears to be true independent of health system contexts. The successful development and implementation of systems will depend on the degree to which these dimensions can be addressed in system design and central and local implementation strategies. We have provided insights into how this may be achieved, through for example, educational strategies, allowing local configurations to emerge, and by considering organisational drivers. These will now inform the development of the MyPath intervention.

In the complex and demanding environment of cancer care, new interventions will only be adopted if they are perceived to add value to patient care without negatively impacting patients or professionals.

## Electronic Supplementary Material

Below is the link to the electronic supplementary material.

**Supplementary Material 1**: COREQ checklist

## Data Availability

No datasets were generated or analysed during the current study.
