# Supplementary material for: Exploring perceptions of digital patient reported outcome measures: a multi-site qualitative case study in four European oncology outpatient clinics
Source: Health Qual Life Outcomes. 2026 Jun 19;24:113. doi: 10.1186/s12955-026-02574-0 (PMC13548537; doi:10.1186/s12955-026-02574-0)
Supplement: Supplementary file 1 [file 12955_2026_2574_MOESM1_ESM.docx]

COREQ (Consolidated Criteria for Reporting Qualitative Research)

Domain 1: Research team and reflexivity

1. Interviewer/facilitator
   Interviews were conducted by trained qualitative researchers at each participating site.
2. Credentials
   Researchers held postgraduate qualifications and/or had experience in qualitative health services and implementation research.
3. Occupation
   Researchers were employed as academic researchers, clinicians, or health services researchers within participating institutions.
4. Gender
   Researchers included both male and female interviewers.
5. Experience and training
   Researchers had previous experience conducting qualitative interviews and received project-specific training and guidance prior to data collection.
6. Relationship established
   In some sites, researchers had existing professional relationships with clinical teams. No prior relationship existed with most patient and caregiver participants.
7. Participant knowledge of interviewer
   Participants received a participant information sheet explaining the aims of the study, the role of the researchers, and the wider MyPath project.
8. Interviewer characteristics
   Researchers adopted a reflexive approach and discussed emerging findings during regular cross-site meetings. The multidisciplinary team included expertise in digital health, palliative care, oncology, implementation science, and qualitative methods.

Domain 2: Study design

9. Methodological orientation and theory
   A qualitative multi-site case study design was employed. Data were analysed using a combined inductive–deductive thematic analysis informed by the Technology, People, Organisations and Macroenvironmental factors (TPOM) framework.
10. Sampling
    Purposive sampling supplemented by snowball sampling.
11. Method of approach
    Healthcare professionals were approached through site gatekeepers. Patients and caregivers were informed about the study through clinical teams and contacted researchers directly if interested.
12. Sample size
    94
13. Non-participation
    Not systematically recorded
14. Setting of data collection
    Interviews were conducted online or in person depending on site preference and participant availability. Observations were conducted in clinical, multidisciplinary team, and implementation planning settings.
15. Presence of non-participants
    Where relevant, caregivers were present during some patient interviews and consultations observed.
16. Description of sample
    Participant demographics and characteristics are presented in Table 4.
17. Interview guide
    Semi-structured interview guides were developed by the multidisciplinary research team and refined iteratively throughout data collection. Guides were not formally piloted with patients or caregivers.
18. Repeat interviews
    No repeat interviews were conducted.
19. Audio/visual recording
    Interviews were audio-recorded with participant consent.
20. Field notes
    Field notes were taken during interviews and observations.
21. Duration
    Interview and observation durations are reported in the Results/Tables.
22. Data saturation
    Formal saturation was not assessed. Recruitment was constrained by project timelines, site capacity, and participant availability.
23. Transcripts returned
    Transcripts were not returned to participants for comment.

Domain 3: Analysis and findings

24. Number of data coders
    Coding was undertaken by local research teams with cross-site discussion of coding frameworks and emerging themes.
25. Description of coding tree
    A coding framework informed by TPOM was developed and refined iteratively.
26. Derivation of themes
    Themes were derived through a combination of deductive coding informed by TPOM and inductive identification of emergent themes.
27. Software
    NVivo was used for data management and coding.
28. Participant checking
    Participants did not review findings.
29. Quotations presented
    Illustrative quotations are provided throughout the Results section and are identified by site and participant role.
30. Data and findings consistent
    Findings are supported by participant quotations and observational data.
31. Clarity of major themes
    Major themes are presented in the Results section and summarised in Table 6.
32. Clarity of minor themes
    Sub-themes and divergent perspectives are reported where relevant.
